# Capacities of women and men to improve maternal and newborn health: Effect of a community-based intervention package in rural Bangladesh

**DOI:** 10.7189/jogh.09.010413

**Published:** 2019-06

**Authors:** Ahmed Ehsanur Rahman, Janet Perkins, Tapas Mazumder, Mohammad Rifat Haider, Abu Bakkar Siddique, Cecilia Capello, Carlo Santarelli, Shams El Arifeen

**Affiliations:** 1 International Centre for Diarrhoeal Disease Research, Bangladesh (icddr,b), Mohakhali, Dhaka, Bangladesh; 2 Enfants du Monde (EdM), Geneva, Switzerland; * Joint first authors with equal contributions

## Abstract

**Background:**

Despite improvement in recent decades, maternal and newborn mortality in Bangladesh remain high. A community-based intervention package was initiated in 2009 in Netrokona, a rural district in Bangladesh, to engage individuals, families and communities to improve maternal and newborn health. In this article, we present the effect of the intervention package on improvement of women’s capacities with regard to maternal and newborn health, their husbands’ capacities to effectively support them and use of skilled services during pregnancy, childbirth and after childbirth.

**Methods:**

We adopted a quasi-experimental design to evaluate the effect of the intervention package. A cross-sectional household survey was conducted in the intervention and comparison sites at baseline in 2014 and at endline in 2016. A total of 725 women were interviewed at baseline (intervention n = 444; comparison n = 281) and 737 at end-line (intervention n = 442; comparison n = 295). A total of 317 of their husbands were interviewed at baseline (intervention n = 178; comparison n = 139) and 731 at endline (intervention n = 440; comparison n = 291). Propensity score matching (1:1) was performed and the subsequent analysis was restricted among 235 matched women at baseline and 217 matched women at endline. Descriptive analyses were performed for the covariates for matching. Bivariate analyses between baseline and endline were done for reporting women and their husbands’ knowledge regarding pregnancy and childbirth, birth preparedness and complication readiness practices and utilization of health services.

**Results:**

There was significant increase in awareness of danger signs during pregnancy, childbirth and following childbirth among women and their husbands, as well as increase in awareness of rights related to maternal and newborn health. There was also significant increase in birth preparedness and complication readiness practice among pregnant women and their husbands in the intervention site. Regarding use of skilled health services, there was significant increase in early initiation of antenatal care, attending at least one antenatal care contact and attending at least four antenatal care contacts. No notable improvement was observed in giving birth in the presence of skilled attendant or use of postnatal care.

**Conclusions:**

We conclude that the intervention package was effective in building the capacities of women and in engaging their husbands positively in maternal and newborn health. This may have translated into increased use of skilled care during pregnancy.

Despite being mostly preventable, an estimated 303 000 women and 2.7 million newborns continue to die each year due to conditions related to pregnancy and childbirth, with the vast majority of these deaths occurring in low- and middle-income countries [1-5]. Recognition of the global failure to ensure the health and well-being of women and newborns galvanized the international development community to revamp efforts and identify innovative strategies to tackle these challenges. This revolution in approach is reflected in the Global Strategy for Women’s, Children’s and Adolescent Health, which envisions a world in which women and children not only survive, but one in which they are able to thrive in transformed societies and environments, realizing their rights to health and well-being [6,7].

Designed to be coherent with the UN Agenda 2030 and the Sustainable Development Goals (SDGs) [8], the Global Strategy has identified nine priority areas for action designed to act synergistically towards the ‘survive, thrive, transform’ agenda [6]. These action areas include increasing individual capacity, aiming to empower women to participate and act as agents for change, and community participation, encompassing and explicitly calling for the engagement of men. While these actions are universally recognized as important, an evidence gap remains as to how best to operationalize these action areas through participatory approaches to achieve social transformation and how such approaches, in turn, contribute to the well-being of women and children [9].

Similar to many other low- and middle-income countries, Bangladesh has made considerable improvements in maternal and newborn health (MNH) over the past two decades [1,10]; however, the country continues to suffer some of the highest maternal and neonatal mortality rates in the world [10,11]. Acknowledging this high burden, the Government of Bangladesh has endorsed the Global Strategy, and is committed to achieving the SDG targets, including to reduce the maternal mortality ratio to at least as low as 70 per 100 000 live births (from 196 per 100 000 live births in 2016) and the neonatal mortality rate to at least as low as 12 per 1000 live birth by 2030 (from 28 per 1000 live births in 2014) [7,8,10,11]. Reaching these ambitious SDG targets will require the country to significantly accelerate the rate of reduction of maternal and newborn deaths from that which was observed during the Millennium Development Goal (MDG) era. Doing so will necessitate fully leveraging each of the action areas identified in the Global Strategy. This includes developing the individual capacity of women to act as agents of change over their health and that of their children; and engaging families and communities to participate in improving care of women and children and care-seeking practices.

In 2009, a Swiss NGO Enfants du Monde and its local partner, PARI Development Trust, began supporting the Ministry of Health and Family Welfare (MOHFW) to implement a programme for improving MNH in selected areas of Netrokona district in Bangladesh. This programme was based on the World Health Organization’s (WHO) framework for Working with Individuals, Families, and Communities (IFC) to improve MNH, which provides a concrete model and tools for governments and implementing organizations to operationalize health promotion for MNH, thereby building the capacities of women and engaging families and communities in improving MNH. Moreover, the IFC approach also aims to stimulate collaboration between community actors and health services, as well as improvements in health services [12]. In this article, we present the effect of the intervention package on three areas of interest: 1) contributing to increasing the capacities of women with regard to MNH; 2) building the capacities of husbands to effectively engage to support women; and 3) use of skilled MNH services.

## METHODS

### intervention description

The intervention package was implemented in a selected sub-district (locally known as upazila) of Netrokona district, a resource-poor, hard-to-reach area located in the northern part of Bangladesh along the border with India. The intervention package was designed based on the WHO’s IFC framework [12] and was rolled out in Kalmakanda upazila beginning in 2009 [13]. Implementing partners included PARI Development Trust, MOHFW at the district level, and several other local NGOs, including Jatio Tarun Sangha, Garo Baptist Convention/Primary Health Care Project and World Vision Bangladesh.

Specific interventions were identified and co-designed through consultation with programme stakeholders, including local MOHFW managers, NGO implementing partners, as well as community leaders and representatives. The programme was initially implemented in the first phase (2009-2012) in four selected unions (smallest administrative unit in Bangladesh with around 25 000-30 000 population each) of Kalmakanda upazila. During the initial phase, the implementation of the intervention package was undertaken with the local implementing NGO taking the lead and their community health workers playing a crucial role. Over time, their role increasingly evolved into support activities for MOHFW staff working in the area as local public health managers took on increased responsibility for the programme.

Beginning in 2013, the programme was expanded to the four remaining unions of Kalmakanda upazila to cover the entire sub-district and the International Centre for Diarrhoeal Disease Research, Bangladesh (icddr,b) was included to evaluate the effectiveness of the program focusing on these expansion areas. At this time, the implementation strategy also evolved and was aligned with the existing Health, Population and Nutrition Sector Development Program, a sector-wide approach initiated by MOHFW in 2011. As such, the programme aimed to shift the entry point of the intervention package to the government community clinics. In this new phase, the implementing partner NGO assumed a supporting role to support local MOHFW health care providers to take responsibility for delivering the intervention package. MOHFW facility-based health service providers and community-based health workers became the main service providers, while the NGO community health workers were reduced to a total of one per union in 2016 from three to four per union in 2009.

**Figure 1:**
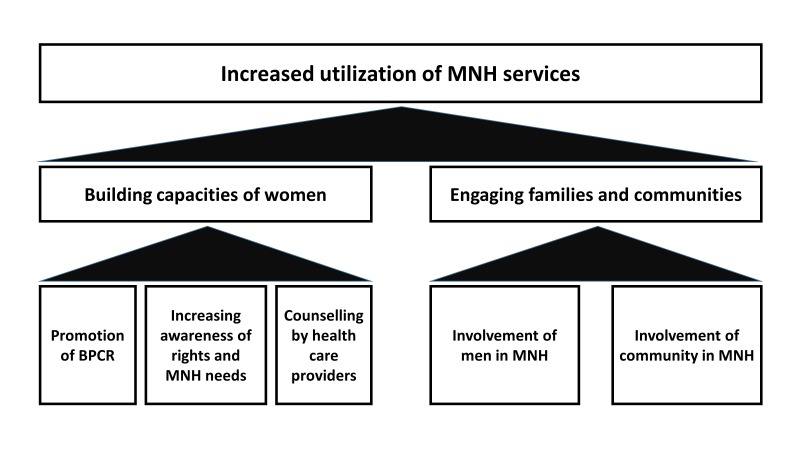
Key interventions and objectives of the intervention package.

Based on the consultative process and the evolution over time of the programme, the following key interventions (most of which were being implemented in the previous interventions unions) were planned and subsequently implemented in the new intervention unions from 2013 onwards to build the capacities of pregnant women and engage communities in MNH (also see **Figure 1**).

### Building individual capacity

The programme aimed to build the capacities of pregnant women through birth preparedness and complication readiness (BPCR), increasing their awareness of the rights and needs associated with MNH, and increasing health care providers’ capacities to effectively counsel pregnant women and their families on MNH care.

#### Promotion of BPCR

The BPCR intervention was co-designed through a consultative process with national technical experts, local MOHFW managers, facility-based health service providers, community-based health workers and representatives from the communities which ensured the reflection of local preferences and context. The BPCR components prioritized through this process were as follows: identification of a desired place for birth, identification of the nearest health facility to consult in the case of complications, identification of a preferred birth attendant, arrangement of transportation to reach the birthplace, saving money to cover potential costs which could be incurred when availing routine and emergency MNH services, and identifying a potential blood donor. This covered six of the eight components recommended by WHO and all of the components promoted by the Bangladesh Maternal Health Strategy [14,15]. In addition, as home birth is still a common practice, arrangement of materials necessary for a safe home birth was included in Kalmakanda. This planning process was conducted in the first phase of the programme in 2009 and the model was used for BPCR implementation in the new intervention unions.

All facility-based health service providers and community-based health workers of MOHFW were trained in BPCR, following which they assisted pregnant women to develop an individualized plan either during ANC contacts in health facilities or through outreach visits in the community. The intervention was supported by a team of community health workers employed by the main implementing NGO.

A card referred to as the “Birth and Emergency Preparedness Plan card”, or “BEPP card”, was developed as a key tool in the intervention package. This BEPP card illustrated the main components of planning for birth and potential complications prioritized by the programme and proposed locally-relevant categories for each component, eg, locally available health services and transport. The card also included illustrations of the most common danger signs in women during pregnancy, labour and after childbirth, as well as danger signs in newborns for which skilled care should immediately be sought.

The BEPP cards were distributed to all identified pregnant women by health service providers during the ANC contacts at health facilities and by community health workers (both government- and NGO-employed) during the scheduled household visits. The BEPP card was used by the facility-based health service providers and community-based health workers to assist pregnant women to develop a plan across the key components of BPCR. If the husband or other family members were present with the woman during the consultation, they were included in developing the plan. Health care providers prioritized decision making by the women during birth preparedness planning. The plan was then reviewed with the woman (along with her husband/family members if present) during each subsequent contact with the health service provider during pregnancy. In addition, she was specifically counselled on key danger signs during pregnancy, childbirth and during the postpartum period, and the importance of seeking care from appropriate health facilities if they experienced any of these danger signs.

#### Increasing awareness of rights and MNH needs

Community-based health workers conducted courtyard meetings with women in the community to discuss rights related to MNH and the special needs of women and newborns during pregnancy, childbirth and after childbirth. The courtyard meeting were gatherings of 20-30 rural people from the community, especially women, their husbands and other family members, organized at a designated place, usually a person’s house in a village. The government community-based health workers, with support from NGO health-workers, organized 1-3 courtyard meetings per community clinics per year. The courtyard meeting usually lasted from half an hour to two hours. Rights topics which were included during these discussions include the right to access MNH services, rights related to decision-making, right to respectful care and right to information. In addition, they discussed the needs of women during pregnancy, childbirth and following childbirth, including BPCR practices and danger signs for which skilled care should be immediately sought.

#### Counselling by health care providers

Finally, the intervention package aimed to build the capacities of facility-based health service providers and community-based health workers (government) to more effectively counsel pregnant women and their families on MNH and support women to act as agents of change for their health. Health service providers and community-based health workers were trained to improve their communication and counseling skills, and thereby their capacities to respond to the needs of women and families. Specifically, the training focused on moving beyond simply providing information during MNH contacts toward increased responsiveness through active listening, two-way communication, demonstrating respect, pre-assessing and building on the knowledge of service recipients, understanding women’s situation and assisting them to develop and follow through on their plans to improve MNH [14].

### Community participation

The intervention package also aimed to increase the participation of the community, and the involvement of men specifically, in contributing to a supportive environment for MNH.

#### Involvement of men

The intervention package specifically targeted men and aimed to increase their involvement in MNH. This was done through the courtyard meetings conducted by community-based health workers to discuss MNH rights and needs of women during pregnancy and childbirth. During these meetings, the roles and responsibilities of men in MNH were also discussed. This was meant to destigmatize men’s involvement in MNH, which is traditionally relegated to the women’s sphere. In addition, husbands were encouraged to participate in BPCR actions. If a husband was present during a contact with a pregnant woman, the health care provider sought to involve him in BPCR, either in elaborating the plan with the woman or in reviewing the plan if it had already been developed. If the husband was not present, the woman was encouraged to discuss her health and BPCR plan with her husband and invite him to participate in future ANC contacts.

#### Involvement of community

Actions were identified to build awareness of the broader community on needs and rights related to MNH, and to mobilize communities to identify and implement solutions to overcome barriers to accessing MNH services. Courtyard meetings conducted by community-based health workers were used as the main platform for dialoguing with the community to discuss the rights and needs of women related to MNH. In addition, communities were mobilized through courtyard meetings and through meetings with community leaders to address barriers to health services access related to transportation and finances. Through these mobilization efforts, some community groups worked together to mend broken roads or build small roads to connect the health facilities with central roads. Some communities mobilized resources to purchase and manage rickshaw vans (non-motorized three-wheeled vehicles) reserved for transporting women to health facilities for childbirth or to receive emergency obstetric or neonatal services. Other communities generated funds in order to offer support to women with limited financial resources to cover costs related to care-seeking for obstetric and neonatal emergencies.

### Study design

A quasi-experimental design was adopted to evaluate the effect of the intervention package. A cross-sectional household survey was conducted in the intervention and comparison sites at baseline in 2014 and at endline in 2016.

### Study settings

The site of the study was in the Kalmakanda sub-district of Netrokona district in Bangladesh (approximate population 300 000), where the intervention package was implemented. Netrokona is a haor (wetland) area, with weak transportation systems and infrastructure. It is among the 14 lowest-performing districts of Bangladesh with poor coverage of MNH services and high newborn and child mortality rates [13,16]. The intervention sub-district was pre-selected in consultation with the Government of Bangladesh and on account of operational efficiency as the implementing NGO (PARI) was based in that sub-district. An adjacent sub-district (Barhatta) with a total population of around 272 000 [13] was selected as the comparison area in consultation with national and local level health managers, and based on the following criteria: population, household size, female literacy rate, employment rate, Expanded Programme on Immunization (EPI) coverage and presence of special MNH initiatives. In addition, Barhatta did not have any large-scale MNH initiative in place during the programme implementation phase in Kalmakanda.

### Study population

Eligible respondents were married women between 15 and 49 years of age with a history of childbirth in the 12-month period preceding the date of survey. Their husbands were also included as a secondary study population.

### Sample size

The study was designed to evaluate the effect of the intervention package on MNH. Skilled attendance at birth was selected as the primary outcome of interest for the sample size calculation and women with a recent history of childbirth were regarded as the primary study population. As per the Bangladesh Maternal Mortality Survey (BMMS) 2010 report (the last national survey to report district specific estimates in Bangladesh), coverage of birth with a skilled attendant was 15.6% in Netrokona. It was assumed that the intervention package would increase the coverage of birth with a skilled attendant by at least ten percentage points (absolute) between baseline and endline. We also wanted to ensure a higher representation from the intervention site (intervention: comparison = 1.5:1), which would allow us to generate more precise estimates of MNH-related knowledge and practices from the intervention site. The unadjusted sample size was 404 from the intervention site and 269 from the comparison site at 80% power and 5% error probabilities. The sample size was then adjusted for design effect/cluster effect (1.25) and non-response (5%). The final sample size was 425 women with a recent history of childbirth from the intervention site and 283 from the comparison site at baseline and at endline. Based on this sample size, we interviewed 725 women with a recent history of childbirth at baseline (444 from the intervention site and 281 from the comparison site) and 737 at endline (442 from the intervention site and 295 from the comparison site). In addition, as an exploratory component of the study, we approached all husbands of the women interviewed during the survey. A total of 317 husbands were interviewed at baseline (178 from the intervention and 139 from the comparison site) and 731 at endline (440 from the intervention site and 291 from the comparison site). The non-response rate among husbands was 56% at baseline and 1% at endline.

### Sampling

**Figure 2:**
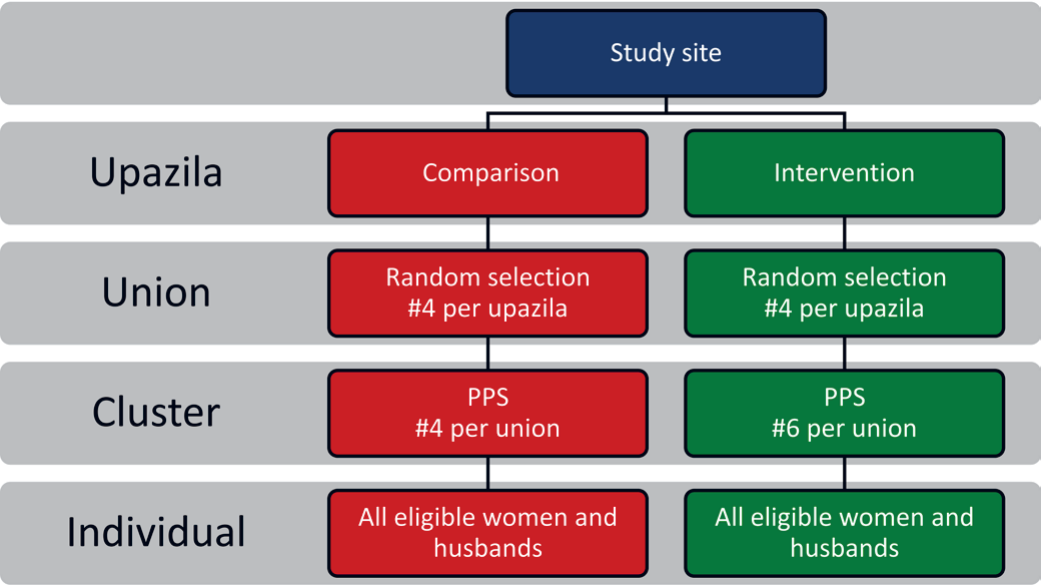
Multi-stage sampling strategy adopted for the household surveys at baseline and at endline.

We adopted a multi-stage cluster sampling technique to select the eligible respondents (**Figure 2**). In the first stage, four unions were randomly selected from each sub-district (intervention and comparison). A total of sixteen villages from the comparison area and 24 villages from the intervention area needed to be selected to reach the required sample size. Therefore, in the second stage, four clusters (average population of approximately 1000) were selected from each of the comparison unions and six clusters were selected from each of the intervention unions using the probability proportional to size (PPS) sampling technique. All eligible respondents from the selected clusters were approached for interviews.

### Data collection

The household surveys were conducted through interviewer-administered questionnaires with structured questions. Most questions were adapted from the Bangladesh Demographic and Health Survey (BDHS) 2011 and the BMMS 2010 [17,18]. Separate questionnaires were developed for women and their husbands. The questionnaire started with questions regarding personal and socioeconomic information followed by questions related to knowledge and practices regarding pregnancy, childbirth and the postpartum period. The questionnaires were pre-tested on women with a recent history of childbirth and their husbands residing in non-selected clusters of the selected unions. Interviewers were recruited locally so that they would be familiar with the local context, culture, and dialect. Adequate training of the data collectors was ensured by experienced trainers and field supervisors. In the first stage of data collection, a sketch map was drawn for each of the selected clusters indicating boundary, landmark, and Bari (household) locations. All households and women who had a birth outcome in the 12 months preceding the survey were enumerated and listed. In the second stage of data collection, all eligible women and their husbands were interviewed with the structured questionnaire. Husbands of these women were interviewed separately after their wives were interviewed by another team of data collectors. Interviewers who interviewed the wives did not disclose that their husbands would also be approached to be interviewed. The baseline survey was conducted in March-May 2014 and the endline survey was conducted in September-October 2016.

### Data analysis

Data analysis was conducted using Stata 13.0 (StataCorp Inc, College Station, TX, USA). We used the standard steps of principal component analysis to generate the socio-economic indices of the households that we have interviewed, based on which the wealth quintile was generated [19,20]. Household-level variables such as household possessions; materials used for the construction of the floor, wall, and roof; drinking water source; toilet facilities; and ownership of land and domestic animals were used to generate this index. Descriptive statistics were used for describing the socio-demographic characteristics of respondents and their knowledge and practices related to pregnancy and childbirth. Categories of knowledge included awareness of women’s rights related to MNH; knowledge of danger signs during pregnancy, childbirth, and following childbirth; and awareness of the need to seek skilled MNH services. Categories of practices included BPCR practices and utilization of skilled MNH services (ANC, birth in the presence of a skilled birth attendant, PNC and care-seeking for obstetric complications).

Since we adopted a quasi-experimental design, we used propensity score matching to match comparison and intervention respondents to create a valid and counterfactual comparison group [21,22]. The propensity score matching (1:1) was performed based on the woman’s age, the woman’s education, employment status, household size, religion, and wealth index. STATA program “psmatch2” was used to perform the matching and after matching the subsequent analysis was restricted among 235 matched women in both comparison and intervention areas in the baseline and 217 matched women in both comparison and intervention area in endline. Descriptive analyses were performed for the covariates for matching. Bivariate analyses between baseline and end-line were done for reporting women and their husbands’ knowledge regarding pregnancy and childbirth, BPCR practices and utilization of MNH services. Difference-in-difference (DiD) analyses were done to report the effect of the intervention package between baseline and endline across comparison and intervention areas. The DiD estimator [(an interaction term of the programme; (0 for comparison and 1 for intervention) and survey period (0 for baseline and 1 for end-line)] shows the true difference between baseline and endline [23]. Linear regression model were fitted with binary outcome variables along with study period (baseline and endline) and arm (intervention and control) of this study to run DiD analyses.

### Ethical approval

Ethical approval to conduct the study in Netrokaona was obtained from the Institutional Review Board of icddr,b (Protocol Number: PR 14024). Written informed consent was obtained from each participant before beginning the interview after they were fully informed as to the objective of the study and the way in which the data would be used. They were also informed that participation was voluntary and could be terminated at any time without reason and without any penalty. Privacy, anonymity and confidentiality of the participants were strictly maintained during data collection and analysis.

## RESULTS

### Background characteristics

**Table 1** summarizes the background characteristics of the survey respondents before and after matching. The majority of the women between the ages of 20 and 34 years and there was no notable difference between comparison and intervention areas and between baseline and endline after matching. At baseline, more than half of the women had no education or less than five years of schooling. At endline, around one-third of the women had less than five years of schooling. The difference between the comparison and intervention areas at baseline and at endline were found to be insignificant. Similarly, around 60% husbands had less than five years of schooling as baseline, with no significant difference between the comparison and intervention arms. In contrast, less than 30% had less than five years of schooling at endline, with a significant difference between comparison and intervention arms. The majority (around 90%) of the survey respondents were Muslim. Around 80% households were in possession of a mobile phone and around 10% owned a television with no difference between comparison and intervention areas. There was also no difference in asset score index between comparison and intervention areas after matching.

**Table 1:** Personal and household characteristics of the survey respondents (after propensity score matching)

	Baseline 2014						Endline 2016					
	**Unmatched**			**Matched**			**Unmatched**			**Matched**		
	**Comp**	**Int**	***P*-value**	**Comp**	**Int**	***P*-value**	**Comp**	**Int**	**P**	**Comp**	**Int**	***P*-value**
	**N = 28)**	**N = 444**		**N = 235**	**N = 235**		**N = 295**	**N = 44)**		**N = 217**	**N = 217**	
	**%**	**%**		**%**	**%**		**%**	**%**		**%**	**%**	
**Age of women (years)**												
15-19	4.6	4.9	0.98	5.5	4.3	0.98	2.0	9.3	<0.01	2.8	8.3	0.84
20-24	36.3	37.8		36.6	38.3		29.8	33.4		34.1	33.2	
25-29	34.9	32.4		34	31.5		42.0	31.3		42.4	29.5	
30-34	17.8	17.3		17.9	18.7		16.3	14.7		12.4	18.9	
35-39	4.3	5.0		3.8	5.1		5.4	7.0		6	8.3	
40-49	2.1	2.4		2.1	2.1		4.4	2.3		2.3	1.8	
**Education of women (years)**												
0-4	53.7	56.5	0.67	51.5	50.2	0.96	19.4	44.1	<0.01	19.4	38.2	0.17
5-9	39.9	38.3		41.3	43.8		71.4	45.4		71.4	47.5	
≥10	6.4	5.2		7.2	6		9.2	10.4		9.2	14.3	
**Religion**												
Other	8.9	8.3	0.79	7.2	6.0	0.58	8.8	12.9	0.08	9.7	11.5	0.53
Muslim	91.1	91.7		92.8	94.0		91.2	87.1		90.3	88.5	
**Household composition (No. members)**												
2-4	30.6	34.0	0.15	29.8	31.9	0.85	33.6	30.3	0.65	38.2	33.6	0.45
5-7	58.7	51.8		57.9	55.3		49.5	51.8		44.7	50.7	
≥8	10.7	14.2		12.3	12.8		16.9	17.9		17.1	15.7	
**Wealth quintile**												
Lowest	15.7	22.8	<0.01	16.6	13.2	0.98	21.7	19.0	0.55	19.8	18.9	0.96
Second	15.0	23.2		16.2	17		16.9	21.9		16.1	16.6	
Middle	18.1	21.2		15.7	16.6		20.7	19.7		20.3	18.9	
Fourth	24.2	17.3		23.8	25.1		20.7	19.5		19.4	20.3	
Highest	27.0	15.5		27.7	28.1		20.0	19.9		24.4	25.3	

### Women’s and their husbands’ knowledge regarding danger signs

**Table 2** presents the knowledge and awareness of women and their husbands regarding danger signs during pregnancy, childbirth and after childbirth, and the importance of care-seeking pactices from skilled providers (unmatched analysis presented in Table S2A in **Online Supplementary Document**). In the intervention area, the percentage of women aware of at least three danger signs during pregnancy increased from 26% at baseline to 83% at endline, with no apparent change in the comparison area (30% at baseline vs 28% at endline). For awareness regarding ≥ three danger signs during pregnancy, there was a significant increase between baseline and endline in the intervention site controlling for the difference between baseline and endline in the comparison site (DiD, *P* < 0.001). Similarly, in the intervention area, the percentage of women aware of at least three danger signs related to childbirth increased from 24% at baseline to 68% at endline. In contrast, the knowledge level did not increase in the comparison area (32% at baseline vs 22% at endline). There was a two-fold increase in the awareness of at least three postpartum danger signs (30% at baseline to 51% at endline) in the intervention area, while the level of knowledge decreased in the comparison site. Regarding newborn-related danger signs, the knowledge of at least three danger signs increased from 63% to 83%, with a declining trend in the comparison area. For all knowledge categories, the intervention had a significant effect (DiD, *P* < 0.001). There was also a substantial increase in husbands’ awareness of at least three danger signs during pregnancy (6% at baseline vs 57% at endline), during childbirth (11% at baseline vs 44% at endline) and after childbirth (27% at baseline vs 77% at endline). In contrast, knowledge remained stagnant in the comparison area between baseline and endline. For all knowledge categories, the intervention had significantly increased levels (DiD, *P* < 0.001).

**Table 2:** Women’s knowledge regarding danger signs related pregnancy, childbirth and after childbirth

Knowledge regarding	Base-Comp	Base-Int	*P*-value	End-Comp	End-Int	*P*-value	DiD (SE)	*P*-value
**(N = 235)**	**(N = 235)**	**(N = 217)**	**(N = 217)**
**%**	**%**	**%**	**%**
**Women**								
Importance of receiving ANC during pregnancy	55.3	71.5	<0.001	42.4	99.1	<0.001	0.41 (0.055)	<0.001
≥3 pregnancy-related danger signs	29.8	26.0	0.355	27.7	82.5	<0.001	0.59 (0.058)	<0.001
≥3 childbirth - related danger signs	31.9	23.4	0.039	22.1	67.7	<0.001	0.54 (0.059)	<0.001
≥3 postpartum danger signs	34.9	30.2	0.279	15.2	51.2	<0.001	0.41 (0.060)	<0.001
≥3 newborn danger signs	69.4	63.4	0.172	41.0	82.0	<0.001	0.47 (0.061)	<0.001
**Husbands**								
Importance of receiving ANC during pregnancy	48.3	77.6	<0.001	34.1	96.3	<0.001	0.33 (0.072)	<0.001
≥3 pregnancy-related danger signs	11.1	5.5	0.030	25.4	56.7	<0.001	0.37 (0.052)	<0.001
≥3 childbirth-related danger signs	9.4	8.1	0.624	21.2	51.6	<0.001	0.32 (0.051)	<0.001
≥3 postpartum danger signs	14.0	10.6	0.262	15.2	44.2	<0.001	0.32 (0.052)	<0.001
≥3 newborn danger signs	30.6	26.4	0.307	36.9	76.5	<0.001	0.44 (0.060)	<0.001

DiD (SE) – difference in difference (standard error)

### Women's and husbands' awareness of rights related to MNH

**Table 3** presents the awareness of women and their husbands regarding women’s rights related to MNH (unmatched analysis presented in Table S3A in **Online Supplementary Document**). Among women, around two-thirds were aware that they have special rights related to pregnancy and childbirth at baseline. At endline, around 98% women were aware of such rights in the intervention area compared to only 40% in the comparison area. The DiD was statistically significant at *P* < 0.001. Similarly, a significant increase was observed regarding the awareness of the right to respectful treatment by health care providers. Less than 10% of women at baseline were aware about their rights to receive information from health care providers in the comparison and intervention areas. However, around one-quarter of women were aware of this right at endline in the intervention area with no apparent change in the comparison area (DID *P* < 0.001). Around 98% women in the intervention area at endline were aware of at least two out of the four key messages related to rights associated with MNH (DiD *P* < 0.001). Similar trends were observed among husbands regarding their awareness of rights related to MNH.

**Table 3:** Women’s and husbands’ awareness regarding MNH rights

Rights related to MNH	Base-Comp	Base-Int	*P*-value	End-Comp	End-Int	*P*-value	DiD (SE)	*P*-value
**(N = 235)**	**(N = 235)**	**(N = 217)**	**(N = 217)**
**%**	**%**	**%**	**%**
**Women**								
Rights related to pregnancy, childbirth and after childbirth	67.2	69.8	0.551	40.1	97.7	<0.001	0.55 (0.055)	<0.001
Right to respectful treatment by health care provider	44.6	33.3	0.047	51.7	61.3	0.126	0.25 (0.084)	0.003
Right to make the decision autonomously to seek services for herself or newborn	3.4	6.1	0.268	21.8	13.2	0.063	-0.09 (0.057)	0.110
Right to information	10.1	4.1	0.043	11.5	26.4	0.005	0.21 (0.054)	<0.001
Mention 2 or more	67.2	69.8	0.551	40.1	97.7	<0.001	0.55 (0.055)	<0.001
**Husbands**								
Rights related to pregnancy, childbirth and after childbirth	64.7	72.2	0.258	38.7	93.0	<0.001	0.47 (0.076)	<0.001
Right to respectful treatment by health care provider	48.5	38.6	0.244	47.6	63.0	0.017	0.26 (0.107)	0.014
Right to make the decision autonomously to seek services for herself or newborn	6.1	4.3	0.640	18.3	9.5	0.039	-0.09 (0.064)	0.175
Right to information	9.1	1.4	0.043	9.8	22.0	0.016	0.20 (0.058)	0.001
Mention 2 or more	28.1	29.8	0.684	37.8	92.2	<0.001	0.53 (0.056)	<0.001

DiD (SE) – difference in difference (standard error)

**Figure 3:**
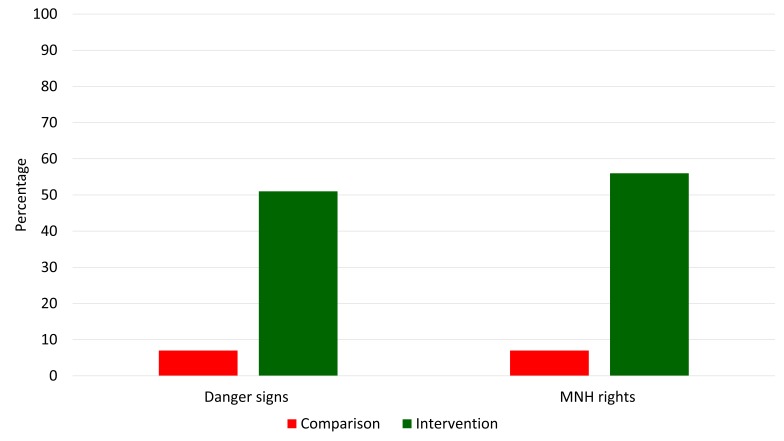
Courtyard meetings mentioned as a source of knowledge and information among women at endline.

**Figure 3** presents the source of knowledge and information among women regarding danger signs and rights relates to MNH. In the intervention area around half of the women reported that they received the information from courtyard meetings compared to less than 10% in the comparison area at endline. Around one-third of the women received the information from government community health workers in the intervention area, whereas less than one-sixth reported government health workers in the comparison area. Similarly, the coverage of NGO recruited community health workers were more than 80% in the intervention area compared to less than 10% in the comparison area. All of these differences (between comparison and intervention for each of the source category) were statistically significant at *P* < 0.001 (Table S3A in **Online Supplementary Document**).

### Birth preparedness and complication readiness

**Figure 4:**
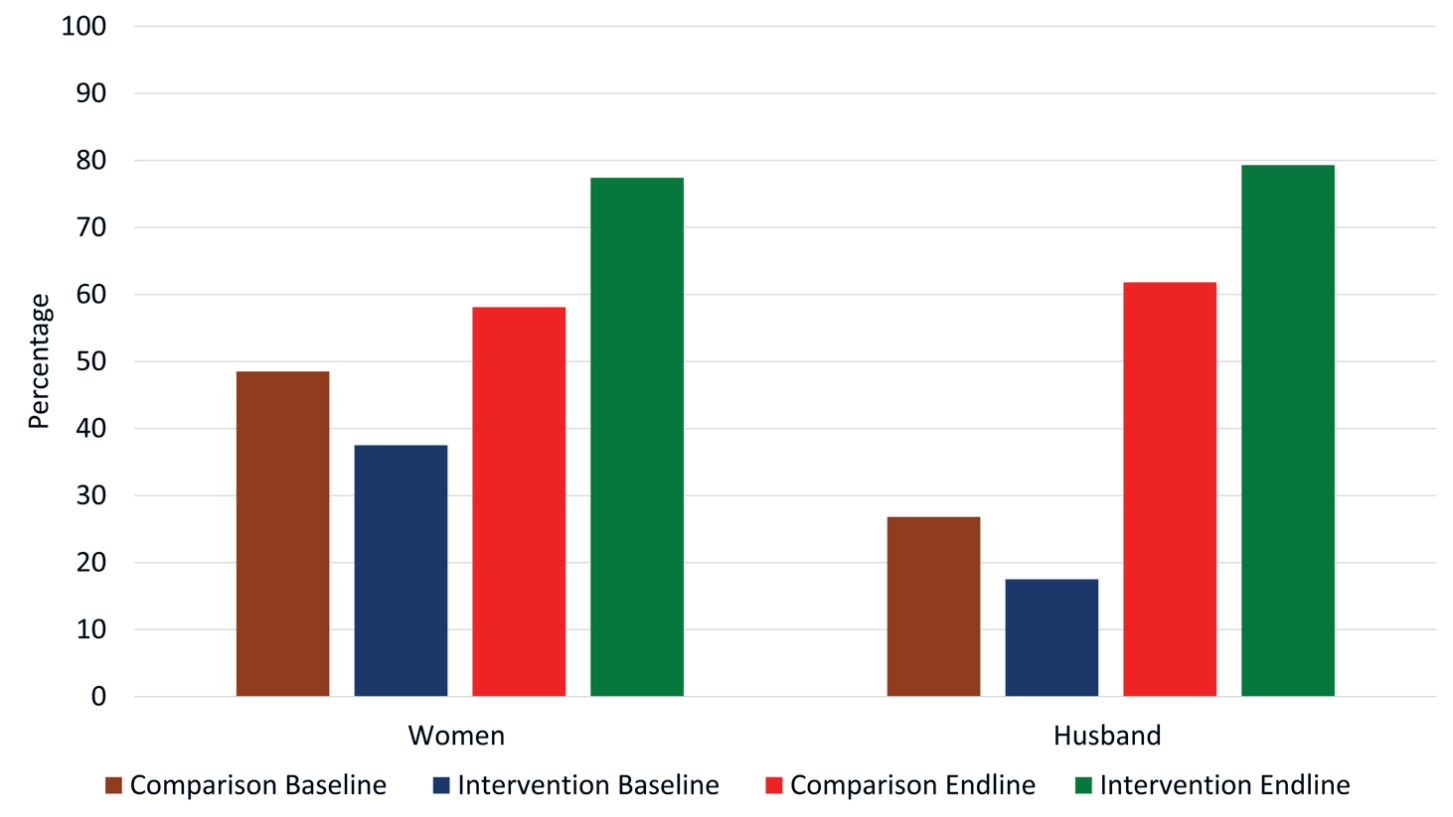
Birth preparedness and complication readiness among women and their husbands; proportions prepared for at least 3 out of 5 components.

We asked the women and husbands regarding their practices related to BPCR. **Figure 4** presents the BPCR practice level (3 or more out of 5 components) among pregnant women and their husbands. The following five key components were considered: selection of a place of childbirth in advance, selection of a birth attendant in advance, arrangement of transportation in advance to reach a health facility, saving additional money for childbirth and emergencies, and identification of a potential blood donor for emergencies. For our anaylsis, we included planning for either facility or home birth as planning this component, as home birth is considered an acceptable option in the Bangladeshi context, provided that a community-based skilled birth attendant (CSBA) (government community health worker trained specifically to manage uncomplicated childbirth) is present. Among women, around 49% in the comparison area and 38% in the intervention area practiced at least three components of birth preparedness at baseline during their pregnancy. However, at endline, around 78% of women had practiced at least three components of birth preparedness in the intervention area compared to 59% in the comparison area. The DiD was statistically significant at *P* < 0.001. Among husbands, around 27% in the comparison area and 18% in the intervention area participated in planning across at least three components of birth preparedness at baseline. However, more husbands in the intervention area had participated in planning three or more or more components of BPCR planning (80%) than the husbands in the comparison area (62%) at endline (DiD *P* < 0.001).

It is worth noting, however, that while planning for a desired place for childbirth and preferred birth attendant was high, a lower percentage of pregnant women planned to give birth in a health facility or with the assistance of a skilled birth attendant at home and the DiD estimator was not significant (baseline comparison, 8.5%; baseline intervention, 5.1%; endline comparison, 13.4%; endline intervention, 8.4%; DiD *P* = 0.709). Less than one-third of the women and less then one-fifth of the husbands mentioned that they discussed BPCR with a health care provider during pregnancy at baseline (no difference between the comparison and intervention area). Although this trend did not change in the comparison area, around three-quarters of the women reported having discussed BPCR with a health care provider during their pregnancy in the intervention area at endline (DiD, *P* < <0.001). Similarly, two-thirds of the husbands reported having engaged in this dialogue in the intervention area at endline (DiD, *P* < <0.001).

### Utilization of health services

**Table 4** presents the utilization of MNH services from skilled health care professionals at baseline and endline (unmatched analysis presented in Tabld S4A in **Online Supplementary Document**). At baseline, less than half of pregnant women received any ANC from a skilled health care professional with no notable difference between the comparison and the intervention area. While this practice remained stagnant in the comparison area at endline, there was a significant increase in the intervention area (87%) (DiD, *P* < 0.001). Similarly, less than one-tenth of pregnant women atended four or more ANC contacts with a skilled health care professional at baseline (no difference between the comparison and the intervention area) which increased to 34% in the intervention area at endline, with no notable increase in the comparison area (DiD, *P* < 0.001). In terms of early initiation of ANC (within the first 12 weeks of pregnancy), there was a significant increase in the intervention area (14% at baseline vs 57% at endline), which remained unchanged in the comparison area (DiD, *P* < 0.001).

**Table 4:** Utilization of MNH services during pregnancy, childbirth, and after childbirth

Birth preparedness	Base-Comp	Base-Int	*P*-value	End-Comp	End-Int	*P*-value	DID (SE)	*P*-value
**(N = 235)**	**(N = 235)**	**(N = 217)**	**(N = 217)**
**%**	**%**	**%**	**%**
**ANC**								
Any ANC from a skilled health care professional	41.0	49.7	0.094	41.5	87.1	<0.001	0.34 (0.067)	<0.001
≥4 ANC from a skilled health care professional	6.4	6.8	0.853	9.7	34.1	<0.001	0.23 (0.044)	<0.001
Received the 1st ANC within first trimester of pregnancy	10.2	14.0	0.203	13.8	57.6	<0.001	0.36 (0.051)	<0.001
**Birth**								
Birth in the presence of a skilled birth attendant (facility or with SBA for home birth)	18.7	14.9	0.267	28.1	19.8	0.043	-0.05 (0.053)	0.323
**PNC**								
Any PNC from a skilled health care professional within two days of childbirth	22.4	16.4	0.111	21.7	21.2	0.907	0.05 (0.055)	0.333

MNH – maternal and neonatal health, ANC – antenatal care, PNC – postnatal care, SBA – skilled birth attendant, DiD (SE) – difference in difference (standard error)

Regarding birth with a skilled birth attendant (either facility-based birth or birth attended by trained provider at home) and PNC from a skilled health care professional within two days of childbirth, there was no notable improvement between baseline and endline in either the comparison or intervention areas.

Among women who experienced complications during their most recent pregnancy, 22% in the comparison area and 33% in the intervention area sought care from medically trained provider at baseline (*P* = 0.164). There was significant difference between the comparison area (41%) and the intervention area (68%) (*P* = 0.047) at endline. However, the difference was not significant after DiD adjustment (*P* = 0.135).

## DISCUSSION

The results of this study indicate that the intervention package was successful in contributing to building the awareness and capacities of women with regard to MNH and to building the awareness and capacities of husbands to positively participate in supporting their wives in MNH. Moreover, our findings indicate that the intervention package contributed to increased use of skilled care during pregnancy. However, it did not result into expected changes in utilization of skilled care during childbirth and after childbirth.

The increase in women’s capacity was reflected in their increased knowledge and improved practices around preparing for childbirth and potential complications. There were significant increases in BPCR practices among women across all components. Our findings are consistent with other studies which have found similar intervention packages to be effective in improving BPCR practices [24-29]. A qualitative study was also conducted in Kalmakanda to explore more in-depth the BPCR component of this intervention package, including barriers and enables within implementation, which has been reported on elsewhere [30]. There were also significant increases along women’s awareness of danger signs during pregnancy, childbirth and after childbirth, which is expected to contribute to their capacity to identify these danger signs in themselves and in their newborns and subsequently seek skilled care. Interestingly, there was a notable increase in women’s education in the comparison site, which was not observed in the intervention site. We are aware that the comparison site did not have any special initiative related to MNH. However, we do not have enough contextual information regarding the on-going education programmes in the comparison site to infer any plausible reason for the observed differences. It can be due to the effect of any education program or a sampling variation/error. It is interesting to note that this increase in education level did not translate into an increase in awareness of danger signs. This also suggests that targeted messaging and counselling around BPCR and danger signs can be effective in promoting awareness of danger signs, irrespective of the educational status. Moreover, we have also controlled for the differences in the individual characteristics while presenting the effect of the intervention package on MNH issues. Therefore, we are confident that such observed differences in individual characteristics did not compromise the validity of our results.

In addition, women’s awareness around rights related to MNH increased significantly in the intervention site. This is important as awareness of rights is requisite to building the capacities of women to claim them. An initial step to this is building awareness that access to the services and conditions necessary for a safe and healthy pregnancy and childbirth experience are rights to which women are entitled. While the dialogue at the global level regarding maternal health has become increasingly centered on rights, few studies have sought to understand the comprehension of rights in relation to MNH or the receptiveness of these concepts at a local level in non-Western countries [31]. The intervention package aimed to increase awareness of these rights among women, their husbands, and the broader community. The results of this study indicate that there was an increase in the awareness of women of these rights. Not only was there a significant increase in the proportion of women aware of rights in general related to MNH, but there were also significant increases in respondents’ awareness of specific rights related to MNH, ie, the right to access MNH services, the right to information and the right to respectful care. Based on these findings, we can assume that the concepts of rights are appropriate for introduction to similar populations and that a targeted intervention package could successfully raise awareness and transform attitudes with regard to rights related to MNH. This is promising, as other research has found that awareness of rights among women is associated with increased utilization of skilled care during pregnancy and childbirth. Notably, a study in India found that informing populations of their entitlements to health service was associated with increased use of ANC and skilled attendance at birth for both low-and middle- to high-caste groups [32].

The study also suggests that the capacities of husbands to be positively involved in MNH increased due to the intervention package. The results demonstrate that male respondents from the intervention site were much more likely to be aware that women have special rights related to MNH and were able to mention more of the specific rights related to MNH. Other studies have found that men’s awareness of rights and gender issues is positively associated with women’s use of ANC and skilled attendance at birth. In China, husbands’ awareness of gender equity has been positively associated with their wives receiving any ANC during pregnancy, the number ANC contacts and childbirth in a health facility [33]. Increased awareness of women’s rights related to MNH among men in the intervention area may have influenced men’s attitudes towards the roles which they should play within MNH. This was reflected in their increased engagement in BPCR practices in the intervention area at endline. Indeed, there was a remarkable increase in husband’s involvement in BPCR planning in the intervention site.

It is also worth noting that there was also an increase in some of the MNH indicators between baseline and endline in the comparison site (eg, increased awareness of men of danger signs, increased awareness of rights in some categories, and increased BPCR practice). This can be explained by the presence of the routine MNH program by MOHFW in the comparison site. However, these improvements were random and had sporadic effect on select number of MNH indicators as the intervention components in the routine program were not comprehensive enough and inefficiently implemented. In contrast, the intervention package implemented in the intervention site had a comprehensive set of interventions targeting a range of MNH domains. The findings presented in our article demonstrates that the intervention package successfully improved MNH across all domains (awareness related to MNH rights, knowledge regarding danger signs, BPCR practices and MNH service utilizations) in the intervention site compared to standard practice. This highlights the gaps in routine programmes and the importance of having a comprehensive package of community-based and facility-based interventions for improving MNH. The alternate explanation could be contamination. However, it is difficult to explain the improvements in selective indicators through the contamination theory.

Finally, the results of our study indicate that the intervention package was effective in improving care-seeking practices during pregnancy. Indeed, a significant increase was detected across all indicators related to ANC: pregnant women attending at least one ANC contact from a skilled health care professional, pregnant women attending at least four ANC contacts with a skilled health care professional and early initiation of ANC. These findings are consistent with other studies assessing BPCR interventions which have overwhelmingly found a link between BPCR and increased utilization of ANC [24-27,29,34-36]. This may suggest that changes in care-seeking behaviour are more easily influenced during the antenatal period and with appropriate planning. In contrast, we did not observe a change in birth with a skilled attendant or in utilization of PNC. Studies to date assessing the relationship between BPCR interventions and skilled attendance at birth have been mixed, with some studies establishing a significant relationship [24,27,34,35,37-40], and others failing to find this correlation [25,28,41-43]. Our study joins those which did not find an increase in use of skilled attendance at birth with a similar intervention package.

In our study, few pregnant women planned to give birth in a health facility in the comparison and intervention areas and this did not change over the intervention period. Therefore, it is perhaps unsurprising that we did not detect an increase in facility childbirth as women were not planning for it. Several factors may be responsible for this. In rural Bangladesh, women have traditionally given birth at home with a traditional birth attendant. The strong socio-cultural norms and preferences during the childbirth and postpartum periods play a particularly instrumental role in determining the care-seeking practice during childbirth and after childbirth [44,45], and these generally change slowly. This may also have been partially due to a weakness in the intervention implementation, in which ANC was prioritized above care seeking for childbirth and during the postnatal period. Moreover, it is important to point out that while facility birth is considered ideal, home birth is still deemed an acceptable option in the presence of a CSBA according to MOHFW guidelines [46]. The recent national survey on maternal mortality in Bangladesh (2016) reported that around half of the deliveries took place at home with wide inequity; ie, 77% among the poorest quintile and 24% the richest quintile [11]. Since Netrokona is one the poorest performing district in respect to maternal and child health indicators and hard-to-reach area of Bangladesh, the coverage of facility-based birth is expected to be low. Although there was a slight increase in planning for home birth with a skilled birth attendant in the intervention area at endline, it did not result into increasing the coverage of skilled attendance among childbirths occurring at home. This implies that pregnant women were often not able to carry through on their plan to have a home birth attended by a skilled attendant, likely due to the limited availability of CSBAs in the intervention area. Indeed, although a national CSBA program in Bangladesh has been ongoing for the past 10 years, coverage remains low [11], and the country is now moving towards a greater focus on midwifery training [46].

Another explanation could be the service availability and readiness of health facilities to provide skilled attendance at birth and manage obstetric emergencies. As per the last national health facility survey (in 2014), less than 10% public hospitals and facilities have have demonstrated readiness for performing all nine signal functions for providing comprehensive care related to childbirth [47]. The condition is worse in the low-performing districts like Netrokona. It is understandable that the women will not want to give birth in health facilities which lack minimum service availability and readiness related to proper care during childbirth [44]. This may also explain the lack of change in care-seeking behaviour for obstetric emergencies. Financial concerns have also been identified as barriers in low-resource settings, which may also be playing a role in preventing utilization of skilled health services in Netrokona [48,49]. While one component of the intervention package was community participation to address financial and transport barriers to health services, we were not able to assess the impact of these actions through this study.

Moreover, we did not detect an increase in care-seeking after childbirth or for routine PNC. Again, this can likely be explained by socio-cultural factors, as the common practice is to limit mobility of women who have recently given birth and their newborns to within the home for approximately a month following childbirth [50]. It is worth highlighting here the short timeframe of the study period (approximately two years between baseline and endline). Given this, it is not necessarily surprising that less change was observed in birth in the presence of a skilled attendant and postnatal care as practices around these periods may be more heavily dictated by a social and cultural norms, therefore more time would be required to affect change on them [44]. Future research should explore birthplace preferences in order to understand the factors contributing to low use of skilled services during these periods; and how facility birth and postnatal services could become more acceptable to women and families.

### Strengths and limitations

It is important to point out some of the limitations and strengths of this study. One of the limitations is the inability to attribute the change in knowledge, awareness, and practice related to MNH to individual components in the intervention package. However, we can attribute some of the changes that we have observed over the intervention period to the total intervention package. Since there is a dearth of evidence regarding operationalization of the WHO IFC framework for a district model of implementation, the novel attempt of developing an intervention package based on this framework and implementing it through routine systems and structures can be regarded as a major strength of our study. Another limitation of the study is the lack of randomization of the intervention clusters. However, we tried to address this by conducting propensity score matching between women from comparison and intervention areas based on their background characteristics. Therefore, the results demonstrating the effect of the program are adjusted for the co-variate differences between comparison and intervention, which adds further strength and more validity to the study findings. We also acknowledge the limitations regarding recall bias and reporting errors. However, we believe that these will not have any impact on the results of the study (difference between comparison and intervention) as the biases would likely be similar with respondents from the comparison and intervention areas. Moreover, we have considered a recall of only 12 months for assessing the knowledge, awareness, and practice related to MNH, whereas other globally and nationally accepted surveys consider three years of recall for such assessments [11,17]. This also adds more validity to the recall-information and study findings. We acknowledge the potential of social desirability bias in our study. We tried to address this by employing data collectors from local communities who are familiar with the local dialect and culture. For further strengthening the study design, we also interviewed the women and their husbands separately with separate sets of data collectors at separate times. The husbands and their interviewers were not informed of the interview outcomes or details regarding the interviews of their wives. Although, the high response rate among women (>98%) can be regarded as a strength of our study, the response rate among husbands was relatively low at baseline (44%). The majority of them were not present during the time of visit (three attempts were made per husband) as they used often live outside of the household for work related issues. To address this, the endline survey was conducted in and around one of the biggest national festivals where the majority of the husbands came back to their homes to celebrate the holidays with their families. Therefore, the non-response rate among husbands was less than 1% at endline. The high non-response rate among husbands at baseline did not have any compromising effect on the results since there was no difference between background characteristics of the husbands who were successfully interviewed and those who were not.

In addition, our study was designed only to measure change among women, while we know that household members (ie, mothers-in-law, grandmothers, fathers-in-law, etc) are also important gate-keepers within the household. However, we have included husbands in our study and presented their knowledge and involvement in MNH; which is one of the important strengths of the study. We were also unable to measure change at the community level, which was an important focus of the intervention package. Moreover, we were not able to assess programmatic aspects of implementation in this study since the evaluation strategy was not designed to address that. Indeed, the high number of partners collaborating in the programme introduced a level of complexity to implementation which may have influenced results.

A final limitation of our study was the short period of implementation of between baseline and endline. Social transformation is a long-term process which requires a significant amount of time to affect change and be able to detect it. This may be one explanation for the limited change in use of skilled health services for childbirth and postpartum.

## CONCLUSION

The results of our study suggest that the intervention package based on the WHO IFC franework was effective in building the capacities of women and in engaging men positively and in MNH. This may have translated into increase use of skilled care during pregnancy. At the policy level, actions to increase the individual capacity of women to act to improve their health and that of their children and to increase the participation of men and the community in MNH should be prioritized along with efforts to increase the provision of skilled MNH services as the country moves forward toward reaching the SDG targets.

## Additional material

Online Supplementary Document
