# Supplementary material for: Capacities of women and men to improve maternal and newborn health: Effect of a community-based intervention package in rural Bangladesh
Source: J Glob Health. 2019 Jan 4;9(1):010413. doi: 10.7189/jogh.09.010413 (PMC6318832; doi:10.7189/jogh.09.010413)
Supplement: Online Supplementary Document [file jogh-09-010413-s001.pdf]

Rahman et al. Capacities of women and men to improve maternal and newborn health: Effect of a community-based intervention package in rural Bangladesh

Table 2A-Women's and Husband's knowledge regarding danger signs related pregnancy, childbirth and after birth (Before Matching)

Knowledge regarding	Base-Comp (N=281) %	Base-Int (N=444) %	P	End-Comp (N=295) %	End-Int (N=442) %	P	DID (SE)	P
**Women**								
Importance of receiving ANC during pregnancy	46.0	74.2	0.000	30.6	94.6	0.000	0.31 (0.061)	0.000
3≥ pregnancy-related danger signs	25.6	22.8	0.376	28.8	80.5	0.000	0.55 (0.050)	0.000
3≥ childbirth-related danger signs	27.4	21.9	0.088	23.1	64.0	0.000	0.50 (0.050)	0.000
3≥ postpartum danger signs	31.0	23.4	0.025	15.3	51.8	0.000	0.40 (0.050)	0.000
3≥ newborn danger signs	63.0	61.5	0.685	40.0	80.8	0.000	0.41 (0.054)	0.000
**Husband**								
Importance of receiving ANC during pregnancy	46.0	74.2	0.000	30.6	94.6	0.000	0.31 (0.061)	0.000
3≥ pregnancy-related danger signs	23.0	16.3	0.132	27.8	56.4	0.000	0.34 (0.060)	0.000
3≥ childbirth-related danger signs	19.4	16.9	0.554	21.7	51.1	0.000	0.29 (0.059)	0.000
3≥ postpartum danger signs	28.8	23.6	0.296	15.5	44.3	0.000	0.31 (0.062)	0.000
3≥ newborn danger signs	61.2	59.6	0.773	38.8	71.6	0.000	0.35 (0.069)	0.000

Table 3A- Women's and husbands' awareness and attitude regarding MNH rights (Before Matching)

Rights related to MNH	Base-Comp (N=281) %	t (N=444) %	P	End-Comp (N=217) %	End-Int (N=217) %	P	DID (SE)	P
**Women**								
Rights related to pregnancy, child birth and after birth	67.2	68.1	0.817	38.1	96.4	0.000	0.57 (0.051)	0.000
Right to respectful treatment by health care provider	44.6	32.8	0.018	52.7	60.6	0.131	0.21 (0.080)	0.009
Right to make the decision autonomously to seek services for herself or newborn	3.4	5.9	0.258	22.3	11.0	0.002	-0.12 (0.053)	0.029
Right to information	10.1	3.2	0.004	10.7	22.8	0.005	0.23 (0.051)	0.000
Mention 2 or more	56.2	63.5	0.050	38.0	96.4	0.000	0.51 (0.051)	0.000
**Husband**								
Rights related to pregnancy, child birth and after birth	63.7	73.8	0.062	36.8	88.4	0.000	0.41 (0.066)	0.000
Right to respectful treatment by health care provider	50.6	38.6	0.090	51.9	60.0	0.131	0.25 (0.096)	0.009
Right to make the decision autonomously to seek services for herself or newborn	5.1	5.5	0.889	17.0	9.0	0.019	-0.09 (0.056)	0.100
Right to information	8.9	1.6	0.013	8.5	20.4	0.005	0.18 (0.054)	0.001
Mention 2 or more	28.1	28.6	0.887	35.9	87.8	0.000	0.52 (0.050)	0.000

Table 4A-Utilization of MNH services during pregnancy, childbirth, and after birth (Before matching)

**Birth preparedness**	**Base-Comp**	**Base-Int**	**P**	**End-Comp**	**End-Int**	**P**	**DID (SE)**	**P**
	**(N=281)**	**(N=444)**		**(N=295)**	**(N=442)**			
	**%**	**%**		**%**	**%**			
**ANC**								
Any ANC from a skilled healthcare professional	42.5	47.0	0.293	35.6	83.6	0.000	0.37 (0.057)	0.000
≥4 ANC from a skilled healthcare professional	6.4	5.4	0.574	8.5	28.5	0.000	0.20 (0.036)	0.000
Received the 1st ANC within 4 months of pregnancy	10.7	12.8	0.383	11.2	53.6	0.000	0.37 (0.042)	0.000
**Birth**								
Birth in the presence of a skilled birth attendant (facility or with SBA for home birth)	18.2	14.2	0.154	24.1	20.1	0.205	-0.07 (0.044)	0.117
**PNC**								
Any PNC from a skilled healthcare professional within two days of birth	19.8	14.4	0.056	18.0	18.8	0.770	0.02 (0.043)	0.609
